# Effect of forced-air warming by an underbody blanket on end-of-surgery hypothermia: a propensity score-matched analysis of 5063 patients

**DOI:** 10.1186/s12871-019-0724-8

**Published:** 2019-04-09

**Authors:** Hiroshi Sumida, Shigekazu Sugino, Norifumi Kuratani, Daisuke Konno, Jun-ichi Hasegawa, Masanori Yamauchi

**Affiliations:** 1 Department of Anesthesiology and Perioperative Medicine, Tohoku University School of Medicine, 2-1, Seiryo-machi, Aoba-ku, Sendai, Miyagi 980-8575 Japan; 2 Department of Anesthesia, Saitama Children’s Medical Center, 1-2, Shin-toshin, Chuo-ku, Saitama City, Saitama 330-8777 Japan; 3 Department of Anesthesia, Katta General Hospital, 36 Shimoharaoki, Kuramoto, Fukuoka, Shiroishi, Miyagi 989-0231 Japan

**Keywords:** Forced-air warming, Underbody blanket, Propensity score matching, Anesthesia information management system, Body temperature, Intraoperative hypothermia

## Abstract

**Background:**

Underbody blankets have recently been launched and are used by anesthesiologists for surgical patients. However, the forced-air warming effect of underbody blankets is still controversial. The aim of this study was to determine the effect of forced-air warming by an underbody blanket on body temperature in anesthetized patients.

**Methods:**

We retrospectively analyzed 5063 surgical patients. We used propensity score matching to reduce the bias caused by a lack of randomization. After propensity score matching, the change in body temperature from before to after surgery was compared between patients who used underbody blankets (Under group) and those who used other types of warming blankets (Control group). The incidence of hypothermia (i.e., body temperature < 36.0 °C at the end of surgery) was compared between the two groups. A *p* value < 0.05 was considered to indicate statistical significance.

**Results:**

We obtained 489 propensity score-matched pairs of patients from the two groups, of whom 33 and 63 had hypothermia in the Under and Control groups, respectively (odds ratio: 0.49, 95% confidence interval: 0.31–0.76, *p* = 0.0013).

**Conclusions:**

The present study suggests that the underbody blanket may help reduce the incidence of intraoperative hypothermia and may be more efficient in warming anesthetized patients compared with other types of warming blankets.

**Trial registration:**

UMIN Clinical Trials Registry (Identifier: UMIN000022909; retrospectively registered on June 27, 2016).

## Background

Forced-air warming plays a critical role in warming patients during surgery [1–3]. This active warming prevents postoperative complications, such as cardiovascular [4] and major bleeding events [5, 6], and decreases the recovery time [7], hospital costs [8, 9], length of hospital stay [8, 9], and mortality [8]. Recent international guidelines (e.g., CG65 of the National Institute for Health and Care Excellence in the UK) strongly recommend use of a forced-air warming device from the time of anesthetic induction to maintain a patient temperature of at least 36.5°C [10, 11]. Efficient perioperative forced-air warming is achieved by convection of warmed air flow [12]. This effect depends on the difference between skin and ambient temperatures and the area of air flow at the skin surface [12, 13]. However, conventional forced-air warming using an over (full) body blanket cannot fully warm the entire body except during cranial or ear, nose, and throat surgery. Thus, upper or lower body blankets are typically used despite being approximately half as effective [14].

Underbody blankets have recently been launched in the market. As these blankets are more expensive than conventional warming devices (e.g., overbody blankets or thermal mattresses with circulating water), the underbody blanket is still not popular. However, underbody blankets, together with surgical draping, enable efficient convection of airflow over the body. This warmed tent produces a larger body surface area that can be warmed by the blanket [15]. Although several prospective studies have recently reported that the underbody blanket is superior to the overbody blanket in preventing intraoperative hypothermia [16–18], its usefulness remains to be elucidated [19]. Those previous studies partially showed the efficacy of underbody blankets but under limited conditions: cardiac and abdominal surgeries. The ultimate aim of this study was to determine the effect of forced-air warming by underbody blankets in statistically matched patients undergoing different types of surgery.

## Methods

This study was approved by the Institutional Review Board and Ethics Committee of Tohoku University School of Medicine (#2015-1-787, approved on March 17, 2016). We applied opt-out consent according to the recruitments of the human research ethics committees at the institutions and local law.

### Study population

**Fig. 1:**
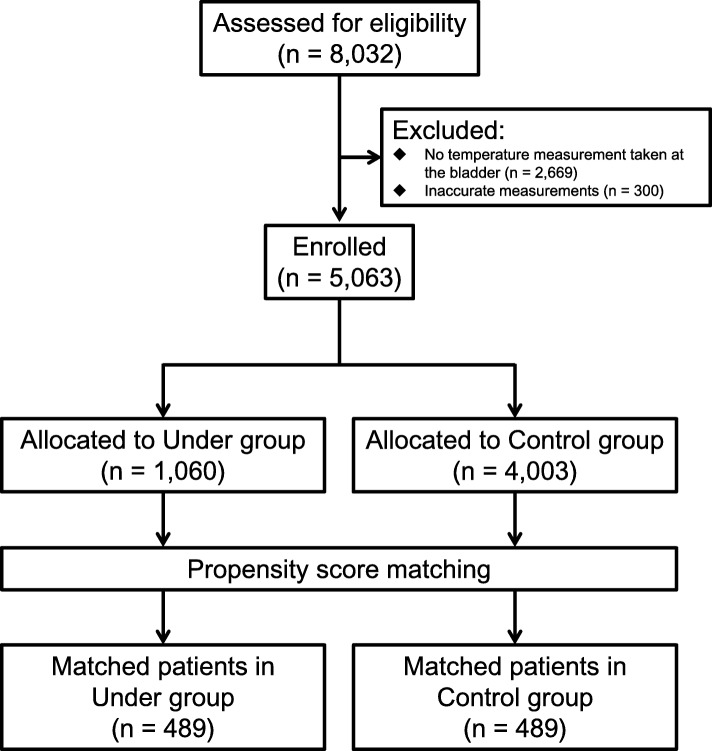
Patient flow diagram

We retrospectively reviewed 8032 consecutive adult patients who underwent surgery in the operating room of Tohoku University Hospital between April 2014 and November 2015. Of these patients, 2669 whose body temperature at the bladder during surgery was not measured (e.g., for surgeries less than 1 h in duration) and 300 with inaccurate body temperature measurements during surgery (i.e., measurement of less than 30.0°C) were excluded from the study. The remaining 5063 patients were enrolled in the study (Fig. 1).

### Warming of patients during surgery

After anesthetic induction, we initiated forced-air warming using two types of warming power units: the Bair Hugger Model 775 (3M Company, St. Paul, MN, USA) and Warm Touch 5300A (Medtronic, Minneapolis, MN, USA). We used the power units with one of four types of blankets: under full body (Bair Hugger Models 545, 585, and 635), over full body (Warm Touch Lower Body Blanket), over upper body (Warm Touch Upper Body Blanket), and over lower body (Warm Touch Lower Body Blanket) blankets. We defined each warming method as follows: under full cover (under full body warming by an under full body blanket), over full cover (over full body warming by an over full body blanket), over upper cover (over upper body warming by an over upper body blanket), over lower cover (over lower body warming by an over lower body blanket). In some patients, the over full body blanket was used to cover only the right or left half of the body (over right cover and over left cover, respectively). Resistive heating blankets (SmartCare, Geratherm Medical AG, Geschwenda, Germany) were used during surgeries performed in a bioclean room (over heating cover). Thus, we used the following seven warming methods: under full cover, over full cover, over upper cover, over lower cover, over right cover, over left cover, and over heating cover. Nurses selected these seven warming methods, either alone or in combination, according to the surgical procedure performed and the appropriate patient position.

### Data acquisition

Raw measurements of vital signs, including body temperature, were transferred onto a server (PRIMERGY TX200 S3, 2 Intel Xenon X5335 processors, 2 GB DIMM, 300 GB HDD) and saved in text format. An electronic anesthesia recording system (PrimeGaia, Nihon Kohden, Tokyo, Japan) was used to retrieve the data from the server at 2.5-min intervals and to display them, together with the patient’s medical information obtained from the hospital information system, as an anesthesia record. Background information on age, sex, height, weight, American Society of Anesthesiology physical status, and type of surgery was obtained. All surgeries were classified into 11 categories defined by the Japanese Society of Anesthesiologists Committee on Operating Room Safety for closed claim studies [20]. The duration of anesthesia from start to end, method of warming (e.g., under full body, over lower body), and body temperature measurement at the bladder were obtained in CSV format from the data warehouse on the server. We assigned the patients warmed by the under full cover method to the Under group and those warmed by the other methods (i.e., all other patients except for those in the Under group) to the Control group (Fig. 1).

### Statistical analysis

We used propensity score matching to reduce potential bias caused by the lack of randomization in this study. The probability (from 0 to 1) of allocation of use of the under full cover was estimated in each patient as a propensity score, based on a multivariate logistic regression model incorporating age, sex, height, weight, American Society of Anesthesiology physical status, type of surgery, duration of anesthesia, and use of one of the other six cover methods (excluding under full cover) as variables. Propensity score matching was performed by random selection of a patient in the Under group and identifying the patient who had the closest propensity score (within 0.03 on a scale of 0 to 1) in the Control group, as described previously [21]. Both before and after propensity score matching, numerical data such as body temperature were compared between the Under and Control groups using Student’s t-test or the Mann–Whitney test. Categorical data were compared between the two groups using the chi square test. The primary endpoint was the incidence of hypothermia at the end of surgery (i.e., a body temperature < 36.0°C, defined by the National Institute for Health and Care Excellence guidelines [10]), which was compared between the two groups using the chi square test. All statistical calculations were performed using SPSS software (ver. 22, IBM Corp., Chicago, IL, USA). A *p* value < 0.05 was considered to indicate statistical significance.

## Results

Table 1 summarizes the demographic and clinical characteristics of the patients in both the Under and Control groups before propensity score matching. Figure 1 shows a flow diagram of the selection of patients into the current study. We obtained 489 propensity score-matched pairs between the Under and Control groups. Table 2 summarizes the demographic and clinical characteristics of the patients in both groups after propensity score matching. The baseline characteristics were not significantly different between the two groups.

**Table 1:** Characteristics of the patients before propensity score matching

	Under group	Control group	Standardized difference^a^	*P* value
Number	1060	4003		
Age (years)	57 ± 21	54 ± 21	14%	< 0.0001
Male / female	521 / 539	2054 / 1949		0.211
Height (cm)	155 ± 21	159 ± 14	19%	< 0.0001
Weight (kg)	55 ± 17	59 ± 17	24%	< 0.0001
ASA-PS				< 0.0001
1 /2	175 / 642	1037 / 2176		
3 / 4 / 5	231 / 12 / 0	737 / 51 / 2		
Type of surgery				< 0.0001
Craniotomy	4	330		
ENT	82	1049		
Thoracic	66	205		
Cardiovascular	81	366		
Endovascular aortic repair	72	49		
Abdominal (laparoscopic)	164	404		
Abdominal (non-laparoscopic)	447	654		
Surface of the trunk	41	387		
Orthopedic	23	380		
Spinal	71	74		
Unclassifiable	9	105		
Type of warming method				< 0.0001
Over full cover	37	1333		
Over upper cover	124	1123		
Over lower cover	237	1652		
Over right cover	5	11		
Over left cover	6	17		
Over heating cover	20	263		
Duration of anesthesia (min)	413 ± 231	307 ± 219	46%	< 0.0001

Data are presented as numbers or means ± S.D. ^a^: Standardized difference for a covariate is the mean difference between the groups divided by the S.D., converted into a percentage

**Table 2:** Characteristics of the patients after propensity score matching

	Under group	Control group	Standardized difference^a^	*P* value
Number	489	489		
Age (years)	57 ± 21	57 ± 20	0%	0.861
Male / female	262 / 227	291 / 198		0.061
Height (cm)	158 ± 18	159 ± 15	6%	0.409
Weight (kg)	55 ± 17	57 ± 17	12%	0.779
ASA-PS				0.247
1 /2	95 / 277	78 / 271		
3 / 4 / 5	110 / 7 / 0	129 / 11 / 0		
Type of surgery				0.695
Craniotomy	4	6		
ENT	51	53		
Thoracic	34	41		
Cardiovascular	67	84		
Endovascular aortic repair	17	19		
Abdominal (Laparoscopic)	69	70		
Abdominal (No laparoscopic)	155	143		
Surface of the trunk	33	26		
Orthopedic	21	21		
Spinal	29	22		
Unclassifiable	9	4		
Type of warming method				0.659
Over full cover	32	38		
Over upper cover	112	97		
Over lower cover	172	178		
Over right cover	2	1		
Over left cover	4	6		
Over heating cover	17	20		
Duration of anesthesia (min)	403 ± 222	431 ± 347	8%	0.137

Data are presented as numbers or means ± S.D. ^a^: Standardized difference for a covariate is the mean difference between the groups divided by the S.D., converted into a percentage. The absolute differences in the mean values of the numerical cofounders included in the matching were less than 15% of the standard deviations

**Fig. 2:**
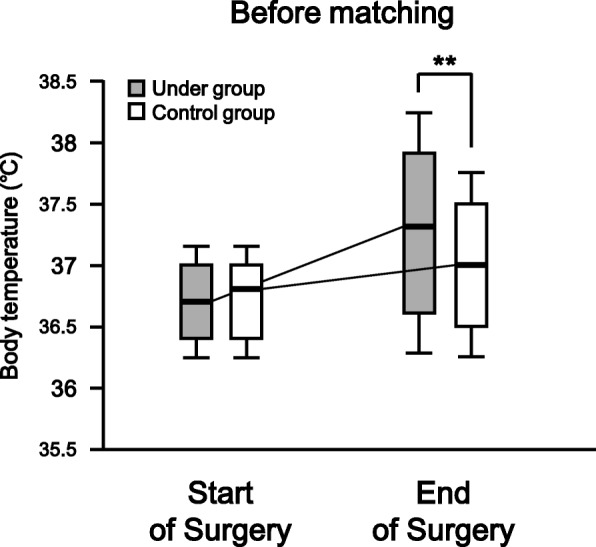
Body temperatures of both groups at the start and end of surgery before propensity score matching. The box and whisker plot shows the median (bold line in box), 25th–75th percentile (top and bottom of the box), and 1.5-fold interquartile range (ends of whiskers) values. **: *P* < 0.01 vs. Control group

**Fig. 3:**
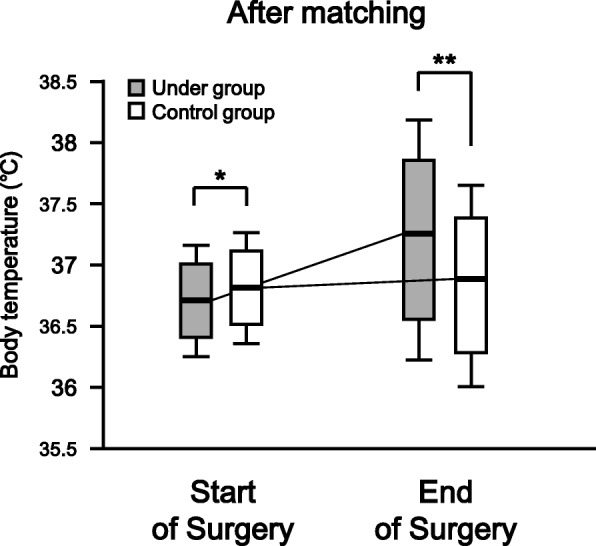
The body temperatures in both groups at the start and end of surgery after propensity score matching. The box and whisker plot shows the median (bold line in box), 25th–75th percentile (top and bottom of the box), and 1.5-fold interquartile range (ends of whiskers) values. *: *P* < 0.05 vs. Control group. **: P < 0.01 vs. Control group

Of the 5063 patients in this study before matching, there were no missing data. The body temperatures [median (interquartile range)] at the start of surgery in the Under and Control groups were 36.7 (0.6)°C and 36.8 (0.6)°C, respectively (*p* = 0.30). In the Under group, the body temperature was 0.6°C higher at the end of surgery compared with the start of surgery. In the Control group, the temperature at the end of surgery increased 0.2°C from that at the start of surgery. There was a significant difference in body temperature at the end of surgery between the Under and Control groups (*p* < 0.0001, Fig. 2). After propensity score matching, 978 total matched patients were evaluated. The median (interquartile range) body temperatures of the matched patients at the start of surgery were 36.7 (0.6) and 36.8 (0.6) in the Under and Control groups, respectively (*p* = 0.04). At the end of surgery, the body temperatures had increased by 0.5°C and 0.1°C from those at the start of surgery in the Under and Control groups, respectively. There was a significant difference in body temperature at the end of surgery between the Under and Control groups (p < 0.0001, Fig. 3).

**Fig. 4:**
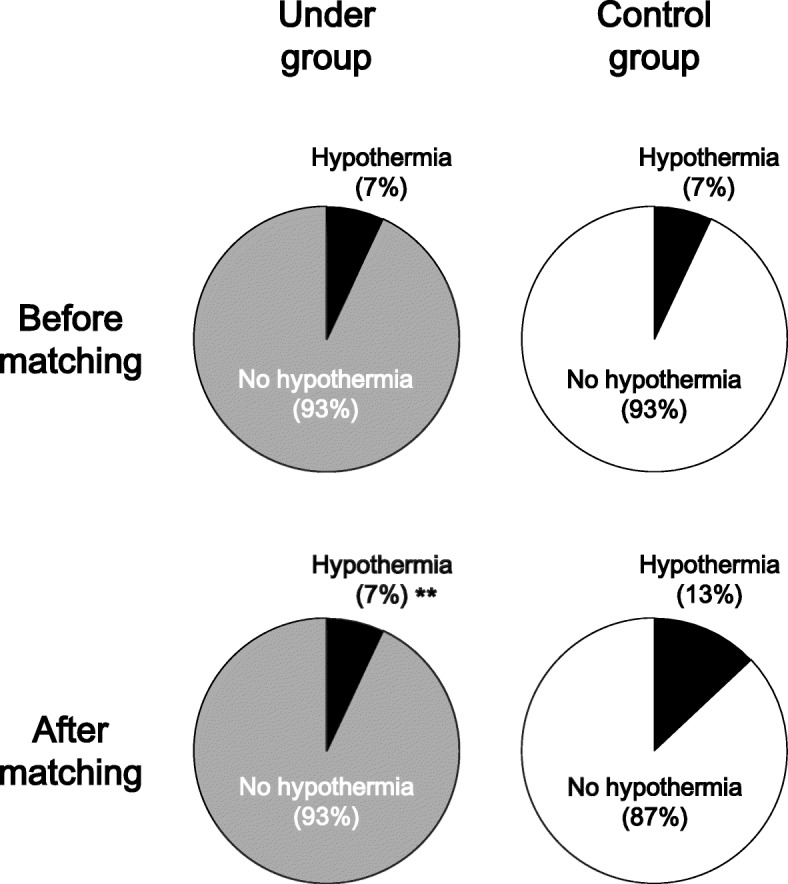
The incidence of hypothermia in the Under (left panels) and Control (right panels) groups before (upper panels) and after (lower panels) propensity score matching. **: P < 0.01 vs. Control group after matching

As shown in Fig. 4, the incidence of hypothermia was identical between the Under (7%, 77/1060) and Control (7%, 274/4003) groups before matching (odds ratio: 1.07, 95% confidence interval: 0.82–1.39, *p* = 0.91). After matching, 33 and 63 patients had hypothermia in the Under and Control groups, respectively, with the incidence of hypothermia being significantly lower in the Under group (7%, 33/489) than in the Control group (13%, 63/489) (odds ratio: 0.49, 95% confidence interval: 0.31–0.76, *p* = 0.0013).

## Discussion

In the present study, we first applied propensity score matching of the patients in the Under and Control groups. We then compared the incidence of intraoperative hypothermia between the two groups. The incidence of hypothermia was significantly lower in the Under group than in the Control group at the end of surgery, as suggested by a significantly higher body temperature at the end of surgery in the Under group compared with the Control group.

A few prospective studies have recently reported the efficacy of underbody blankets in patients undergoing cardiac or abdominal surgery [16–18]. Those reports showed that warming by underbody blankets resulted in an ~ 0.5°C higher body temperature compared with controls, which is consistent with the current results. In the most recent report, however, Alparslan et al. showed that forced-air warming by underbody blankets was as efficient as that by upper body blankets in patients undergoing lower abdominal surgery [19]. The average intraoperative body temperatures of the patients using underbody and upper body blankets were 36.3°C and 36.1°C, respectively. The authors explained that greater heat loss via radiation occurred in the patients warmed by underbody blankets, because the upper frontal body was uncovered in their experimental conditions. However, their comparisons may have been statistically fallacious, because they used the unpaired t-test or Mann–Whitney U-test to compare the change in body temperature at each time point without using repeated-measures analysis of variance. Nevertheless, we agree with their implication that the patient profile and type of surgery performed are limitations in the research on forced-air warming blankets.

In the current study, to reduce the bias caused by the lack of randomization, we applied propensity score matching to the data obtained retrospectively from the electronic medical records of patients who underwent various surgical procedures. The use of propensity scores should be considered when comparing two treatments in an observational design, particularly in cases of highly imbalanced treatment groups, a large number of confounders, or a low number of events [22–24]. Indeed, as shown in Table 1, there were many imbalances between the Under and Control groups, such as the baseline characteristics of the patients, type of surgery, and method of warming. Furthermore, the incidence of hypothermia, the primary endpoint, in each group was low (7% in both groups; Fig. 4). We recognize that the application of propensity score matching in our cohort was reasonable. As shown in Table 2, the matching was considered appropriate, because the standardized differences after matching were less than those before matching by nearly 10%, as was also described previously [24]. Only after matching did we detect a difference in the incidence of hypothermia between the matched cohorts compared with before matching (Fig. 4). In this study, we demonstrated the effects of forced-air warming using an underbody blanket.

Unexpectedly, after matching, the body temperature at the start of surgery showed a tendency to be higher in the Control group than in the Under group, although there was no significant difference between the two groups before matching (before matching: *p* = 0.30; after matching: *p* = 0.04; Figs. 2 and 3). One possible explanation for this difference is that the sex distribution changed after matching (Table 2). In the Control group, the proportions of males and females among the total patients were 51% (2054/4003) and 49% (1949/4003) before matching but 60% (291/489) and 40% (198/489) after matching, respectively. One reason for this is that the number of patients in the Control group who underwent gynecological surgery was lower before compared with after propensity score matching (data not shown). The body temperature of females may be lower than that of males [19, 25], perhaps attributed to the lower skeletal muscle mass of females, which results in a lower basal metabolic rate. However, our subgroup analysis showed no significant difference in body temperature before surgery between the male and female patients of the Control group after matching (data not shown). The underlying mechanism remains unknown under our experimental conditions.

The present study has several important limitations that should be noted. First, the cohort did not include all types of surgery, such as those lasting less than 1 h in duration. Such minor surgeries do not require temperature monitoring at the bladder. This exclusion criterion may have produced selection bias. However, if these patients were included in the analysis, the effect of warming by the underbody blanket would likely have been small. In addition, propensity score matching *per se* may introduce potential selection bias. Although we robustly matched 988 patients using a previously described method [21], the ratio of matched patients to all patients in the Control group was only 12% (489/4003). Furthermore, as described above, many of the patients who underwent gynecological surgery in the Control group were not included in the matched cohort. Thus, in the current study, selection bias may have been present. Second, the anesthesiologists and nurses changed the temperature of the forced airflow according to body temperature values during surgery. This information bias could not be minimized in our study design. In addition, propensity score matching methods ensure balance only of the measured, and not the unmeasured, confounders [24]. The variables that were not measured, such as intraoperative patient position, presence of fever of unknown origin, ambient temperature, or amount of bleeding during surgery, may have influenced the interpretation of the data by introducing information bias. Taking these limitations into consideration, prospective studies with randomization to minimize confounding are needed. In our ongoing trial (UMIN Clinical Trials Registry identifier UMIN000027991), we are comparing body temperatures in female patients undergoing gynecological surgery in the lithotomy position using underbody versus upper body blankets as the warming methods (unpublished).

## Conclusions

The present study suggests that underbody blankets help reduce the incidence of intra-operative hypothermia. The underbody blanket resulted in superior forced-air warming performance compared with the control warming methods.
